# Intratumoral immune-biomarkers and mismatch repair status in leiyomyosarcoma -potential predictive markers for adjuvant treatment: a pilot study

**DOI:** 10.18632/oncotarget.25747

**Published:** 2018-07-20

**Authors:** Jonathan E. Cohen, Feras Eleyan, Aviad Zick, Tamar Peretz, Daniela Katz

**Affiliations:** ^1^ Sharett Institute of Oncology, Hadassah-Hebrew University Medical Center, Jerusalem, Israel; ^2^ Institute of Oncology, Assaf Harofeh Medical Center Zrifin, Beer Yaacov, Israel

**Keywords:** leiomyosarcoma, programed cell death ligand-1, CD8, mismatch repair deficiency, survival

## Abstract

Leiomyosarcoma is the second most frequent soft-tissue sarcoma. Tumor lymphocytic infiltration (TIL) and programed cell death ligand-1 (PD-L1) have been associated with prognosis in different malignancies while DNA mismatch-repair deficiency (MMR-D) has been associated with response to check-point inhibitors. In this pilot study, we sought to examine TIL, PD-L1 and mismatch-repair (MMR) protein expression in 11 leiomyosarcoma and its association with outcome as potential biomarkers for adjuvant treatment.

Eleven primary leiomyosarcoma archived-tissues were analyzed for expression of MMR proteins (MSH2, MLH1, MSH6 and PSM2), PD-L1 expression and PD-1, CD3 or CD8.

MMR-D was detected in tumor tissue from 2/11 leiomyosarcoma patients. CD3 T-cells were present in all samples, whereas CD8 staining was positive in all but one. PDL-1 was positive in 4/11 and PD-L1 in 6/11. Interestingly, the three patients with the poorest outcome had strongly positive staining for PD-L1 and CD8 while in the two patients who are alive and recurrence-free, both PD-L1 and CD8 infiltration were lacking.

We found an association between tumor infiltrating CD8 cytotoxic lymphocytes, strong PD-L1 staining and survival; suggesting a role as biomarkers for treatment decisions regarding peri-operative chemotherapy. We also identified MMR-D in two patients with leiomyosarcoma comprising 18% of our sample.

## INTRODUCTION

Leiomyosarcoma (LMS) is one of the most common histological subtypes of soft tissue sarcoma (STS) with five year disease specific survival for metastatic non resectable disease of 28% [1] and an overall survival of 2.6 years [2]. Given the extremely poor outcome for metastatic patients, peri-operative systemic chemotherapy is being constantly evaluated to reduce recurrence and improve survival, with controversial findings. Results of neo-/adjuvant therapy have been conflicting with seemingly only a small proportion benefiting from it. For example one randomized neoadjuvant trial of ifosfamide, doxorubicin, mesna showed no significant benefit in high risk tumors (over 8 cm of any grade or grade II/III less than 8 cm) in overall survival (OS) or disease free survival (DFS) [3]. In another study with a similar ifosfamide, doxorubicin protocol however there was a disease specific survival benefit in high grade tumors >10 cm but not in smaller tumors with no overall survival benefit [4]. A meta-analysis in the adjuvant setting showed a benefit in recurrence free survival (RFS) and overall survival (odds ratio 0.56) in favor of combination chemotherapy with doxorubicin and ifosfamide [5]. On the other hand, the recent prospective randomized European Organization for Research and Treatment of Cancer (EORTC) 62931 trial investigated adjuvant doxo/ifos vs. observation and found no significant difference in RFS or OS between the groups although there was a trend in favor of the treatment arm in extremity high grade tumors [6].

This marginal benefit is counterbalanced by severe toxicity. For example, in the EORTC trial combination doxo/ifos was associated with 47% grade III/IV toxicity, most commonly neutropenia (39%) [6]. There is currently no predictive biomarker to select patients most likely to benefit or in the highest need for peri-operative chemotherapy.

Immunotherapy in the form of checkpoint inhibitors is revolutionizing cancer therapy with new indications for anti-PD-1, anti PD-L1, anti-CTLA4 and their combinations approved continuously [7]. Immune checkpoint inhibitors allow the recognition of evading tumor cells by antitumor cytotoxic CD8 T cell inducing long-lasting tumor responses. Experience with treatment of soft tissue sarcoma STS with checkpoint inhibitors is limited and initial reports in restricted numbers of STS histologies have been inconclusive with better results in liposarcoma and undifferentiated pleomorphic sarcoma [8, 9]. Attempts are ongoing to identify predictive biomarkers for immune-therapy.

One such candidate biomarker is the expression of PD-L1, an immune check point ligand, expressed by tumors cells or tumor-associated immune cells, acting as an effective tumor mechanism to evade immune-mediated destruction [10]; positive immunostaining correlates with response to anti-PD-1 agents in non-small cell lung cancer (NSCLC) but is not a universal marker in other tumor types [11]. Another important factor with both prognostic and predictive implications is tumor lymphocytic infiltration, specifically that of antitumor CD8 cytotoxic cells [12].

The ability of cytotoxic CD8 positive T cells to recognize tumor cells as foreign and thus mount an immune attack, is also dependent on the presentation of non-self antigens, known as *Neo-antigens* by the tumor. *Neo-antigens* are the result of mutations in coding regions of the DNA, thus tumors with a higher mutation load would be more likely to be identified and destroyed by engaged CD8 T cells once exposed to checkpoint inhibitors [13].

Conditions predisposing to a higher mutational load include internal factors destabilizing the DNA. One such condition is tumors with mutations in the mismatch repair genes. These tumors have two distinguishing characteristics: 1. Microsatellite instability, which is the expansion or reduction in the length of repetitive DNA sequences (known as microsatellites) in the tumor. 2. Loss of one or more of the mismatch repair proteins (MMR-D) in the tumor resulting in a defective apparatus of DNA repair and accumulation of mutations. Recently it has been recognized that irrespective of histology, MMR-D tumors are highly responsive to immunotherapy with the anti-PD-1 checkpoint inhibitor pembrolizumab [14] and MMR-D has recently been approved by the FDA as an indication for pembrolizumab treatment.

This pilot study set out to explore immune-cell infiltration in LMS, its association with DNA mismatch-repair deficiency, microenvironment and clinical outcome. This was undertaken in an effort to explore the immune landscape of LMS and identify potential prognostic markers identifying patients most likely to benefit from neo/adjuvant chemotherapy and immunotherapy, in the form of checkpoint inhibitors. We focused on LMS, a common subtype of STS, and performed our analysis on tissue obtained from the primary tumors to maintain homogeneity.

## RESULTS

### Clinicopathological data

A total of eleven LMS cases were identified. Of these, five were primary uterine ULMS and the remaining were primary extra-uterine LMS (EU-LMS), including testis, dermal, extremity and retroperitoneum). At the time of diagnosis five had a local disease amenable to curative resection while six were metastatic at presentation.

At the time of data collection, four patients were still alive after a mean follow-up of 75 months (range 24–120), two of whom had recurred but were surgically rendered disease free; the remaining seven patients succumbed to their disease with a mean overall survival of 23.8 months (range 4–38).

### Immunohistochemistry staining

**Figure 1:**
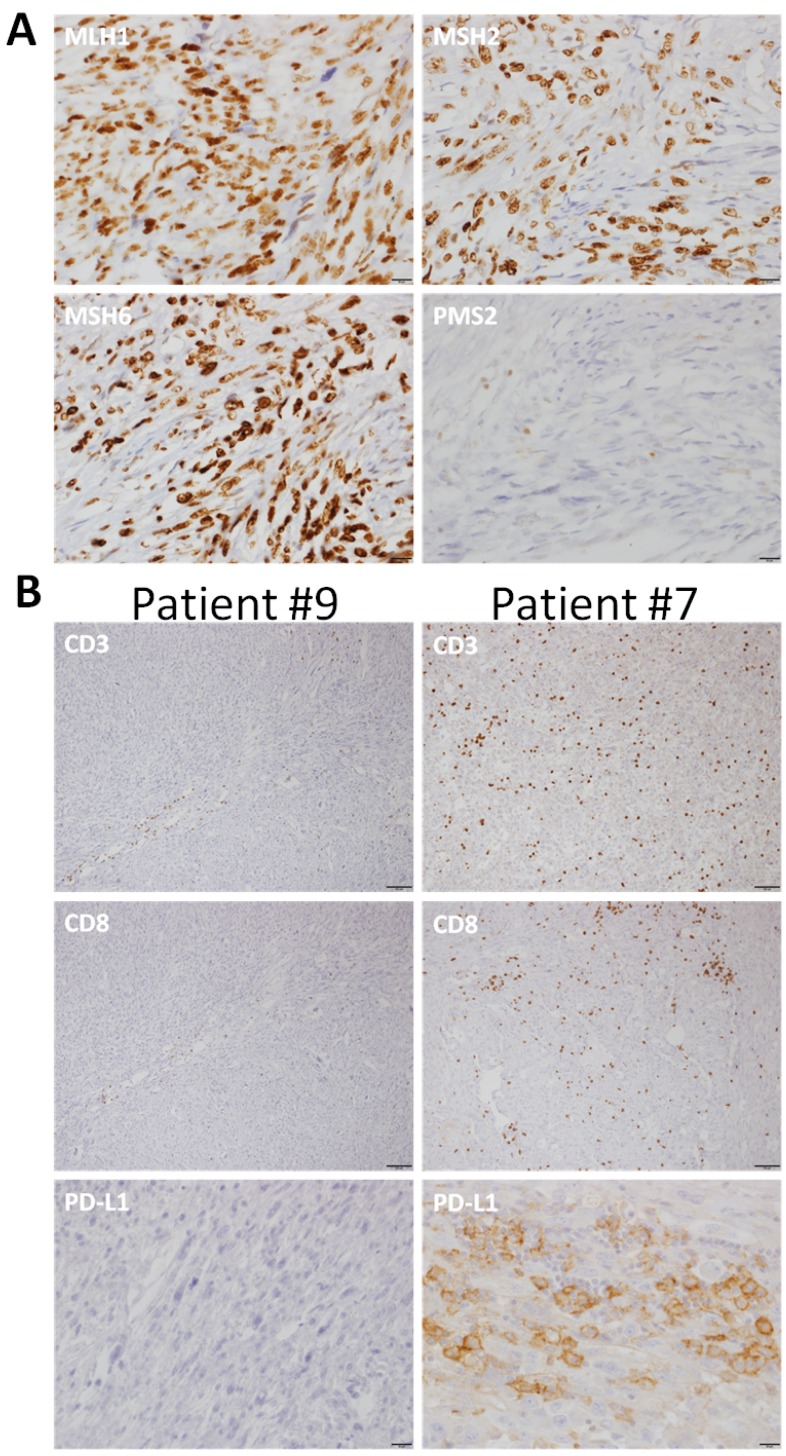
Representative photomicrographs of immunohistochemistry (**A**) Immunostains for the mismatch repair proteins show positive nuclear staining for hMLH1, hMSH2, hMSH6 and no nuclear staining for hPMS2. Note positive internal control for hPMS2 in scattered lymphocytes (bars in A represent 20 μm). (**B**) Immunostains for PD-L1, CD3 and CD8. Patient #9 shows a tumor with negative staining for PD-L1 and low numbers of CD3 and CD8 lymphocytes. Patient #7 shows tumor with PD-L1 positive tumor and immune cells and high numbers of CD3 and CD8 lymphocytes (bars in B represent 100 μm for CD3 and CD8 and 20 μm for PD-L1).

Loss of expression of MMR proteins (MMR-D) was detected in tumor tissue from two of eleven LMS patients (18%, 1 ULMS, 1 EU-LMS). Interestingly, in both cases the deficient protein was PMS2 without associated loss of MLH1 (See Figure 1A).

Tumor infiltrating lymphocytes (TIL) were present in all evaluable samples as evidenced by anti-CD3 staining, in the tumor (11/11) and its periphery (9/9). Cytotoxic CD8 positive cells were identified in 10 of 11 tumors; however, in the tumor periphery these cells were absent in two of 9 evaluable tumors (Table 1 and Figure 1B).

**Table 1:** Expression of immuno-staining and mismatch repair status of 11 primary leiomyosarcomas associated with patient survival

#	Diagnosis	Survival	MMR status	PDL-1 intensity	PDL-1 % positive cells	PD-1 T intensity	PD-1 P intensity	CD3 T intensity	CD3 P intensity	CD8 T intensity	CD8 P intensity
1^*^	LMS extremity	38 m	PMS2 deficient	0	0%	1	2	1	2	1	1
2	ULMS	31 m	Proficient	0	0%	0	0	1	1	2	2
3	LMS extremity	28 m	Proficient	1	20%	1	NA	1	NA	1	NA
4	ULMS	28 m	Proficient	0	0%	1	0	1	NA	1	NA
5	LMS testis	23 m	Proficient	3	1%	0	1	3	1	2	1
6	ULMS	15 m	Proficient	3	5%	2	2	1	1	2	1
7	LMS retroperitoneum	4 m	Proficient	3	5%	3	1	3	3	3	3
8	ULMS	Alive	Proficient	0	0%	3	3	3	3	1	0
9	LMS dermis	Alive	Proficient	0	0%	0	0	1	1	0	0
10	ULMS	Alive (Recurrence free)	Proficient	0	0%	0	0	3	3	2	3
11^*^	ULMS	Alive (Recurrence free)	PMS2 deficient	0	0%	0	0	2	2	1	1

Abbreviations: LMS, leiomyosarcoma, ULMS, uterine Leiomyosarcoma, PD-L1, programmed cell death ligand 1, PD-1, programmed cell death 1, T, tumor, P, tumor periphery. ^*^patients with MMR-D sarcomas.

PD-L1 staining was positive in six of eleven tumors. PDL-1 staining was positive in four of eleven tumors employing the Abcam antibody while all 11 samples had a negative staining with the Dako antibody. Among the two patients with MMR-D, we observed scarce cytotoxic T cell infiltration (+1) and absence of PD-L1 expression.

### Immune-staining-outcome correlates

To explore a potential association between LMS outcome, infiltrating cytotoxic T cells and expression of PD-L1 on tumor cells, we analyzed these parameters for each of the tumors. Among the two patients who are alive and recurrence free, the most favorable subgroup, there was an absence of PD-L1 staining and a lack of peri-tumoral CD8+ infiltration. Conversely, among the three patients with the poorest outcome there was a strongly positive staining for PD-L1 (Abcam antibody) with a positive CD8 staining (Table 1). Interestingly, patients who recurred had peri-tumoral CD8+ infiltration without PD-L1 expression in the tumor.

We did not observe a relation between PD-1 and CD3 staining and outcome. The association between outcome and TIL, PD-L1 and mismatch-repair (MMR) protein expression, was tested using both the Fisher's exact test and the non-parametric Mann-Whitney test. Due to the small sample size, the associations observed were not found to be statistically significant (all *p*-values were > 0.05).

## DISCUSSION

In this report, we sought to link outcome with somatic MMR-D and tumoral immune response as possible biomarkers for prognostic decision regarding peri-operative chemotherapy.

We identified MMR-D in 2 patients with LMS comprising 18% of our sample. Tumor infiltrating cytotoxic CD8 positive cells were identified in all tumors; however, in two these were absent from the tumor periphery. PD-1 and PD-L1 staining were variable; importantly we found an association between CD8 in the periphery, strong PD-L1 staining (Abcam) and clinical course and survival.

MMR-D status is linked to higher number of mutations and the occurrence of *neo-antigens*. The MMR-D status in LMS points to some LMS as potentially sensitive to immunotherapy. The two patients whose tumors were MMR-D had unexpectedly sparse CD8 infiltration and a lack of PD-L1 expression. This is in contrast to reports on epithelial neoplasms where abundant CD8 infiltration was observed in MMR-D tumors such as colon, endometrial and gastric cancers [15–17].

Interestingly, PMS2 was the missing protein in both MMR-D cases. In an analysis of uterine tumors of which 20 were cases of LMS, two were MMR-D (one due to loss of PMS2 the other of MSH2 and 6) [18]. Loss of PMS2 is the least common deficiency identified in MMR-D tumors from various origins, and is associated in most cases with loss of MLH1. Our own results as well as one of the cases reported by Hoang *et al.* suggest the relative excessive frequency of PMS2 loss may not be a coincidental finding as the cause for MMR-D in LMS; however, this remains to be determined in a larger series.

### Cytotoxic CD8 infiltration

All samples in our analysis had CD8+ lymphocytic infiltration within the tumor; however, In our homogenous series of primary LMS, lack of peri-tumoral CD8 infiltration, together with an absence of PD-L1 staining was in fact associated with the best clinical subset- alive and recurrence free in contrast to the patients in our series who had strong positive PD-L1 staining who had the poorest outcome.

The prognostic value of intra-tumor and peri-tumor lymphocytic infiltration has gained attention in recent years. For example, in cutaneous melanoma [19] pancreatic adenocarcinoma [20] and colon cancer [21] peri-tumoral but not intra-tumoral lymphocytic infiltration was a positive prognostic factor.

PD-L1 expression by itself without checkpoint inhibition appears to be a bad prognostic marker; PD-L1 expression has been found in meta-analyses to be a negative prognostic marker such as in non-small cell lung [22], breast [23] cancer and in an analysis across cancer types [24]. In line with these findings, the patients in our series with the poorest outcome had positive PD-L1 staining (Abcam). Results in sarcoma are less conclusive. In a report of 82 patients with STS (non-of whom had LMS), PD-L1 expression was adversely associated with survival; of note PD-L1 expression in this study varied significantly between histological subtypes emphasizing the importance of studying homogenous populations [25]. A similar report comprising 105 patients with STS, 20 of whom had LMS, found a negative association of PD-L1 (clone H-130, Santa Cruz) but also of PD-1 expression on overall survival [26]; whereas in our series which employed different clone of antibodies to PD-L1 only PD-L1 (Abcam) was associated with prognosis but not PD-1. D'angelo *et al.* [27] on the other hand found no association between PD-L1 expression and prognosis in fifty STS patients (clone 5H-1, DAKO). The use of different anti-PD-L1 antibodies along with different methods of interpretation possibly explain part of the discrepancy between PD-L1 expression and outcome in sarcoma [11]. In our own data the Abcam antibody for PD-L1 differentiated between patients while the Dako assay was negative in all patients. In clinical use, specific antibodies (by Dako and Ventana) have been validated as predictive biomarkers for response to anti-PD1 treatment. One possible explanation for the discrepancy between the two antibodies we tested is that the two antibodies have inherently different affinities to PD-L1. Another possibility may relate to PD-L1 having various splice variants with various antibodies preferentially binding different isoforms in an all-or-none fashion or with differing affinities [28]; it may be that the Abcam antibody binds an isoform with prognostic implications not identified by the Dako antibody.

In recent years, the complexity of the immune-response and tumor resistance mechanisms are beginning to unravel. It is instructive to view our own results in this context. Tumors can be categorized as primary, adaptive or acquired immune-resistant or immune-responsive [29]. Contrary to our findings, it might be expected that MMR-D tumors harboring a high mutational load would exhibit a rich lymphocytic infiltration; this discrepancy could be the result of T cell exclusion. A possible mechanism for this primary resistance is loss of PTEN [30] found frequently in LMS [31]. In such a scenario, targeted therapy rather than immune-therapy might restore immune-responsiveness through inhibition of the PI3K-AKT pathway. Our poor prognosis patients had CD8 infiltration but also PD-L1 staining, identifying them as a sub-group with adaptive resistance. This group could potentially benefit from PD-1/PD-L1 blockade restoring immune-responsiveness. Interestingly, the patients with the best outcome in our series lacked both PD-L1 expression and CD8 cells in the peri-tumoral region; this could point to less aggressive biology of the tumor with less turn-over and antigen exposure. This situation may mimic a scenario of a patrolled jail (localized tumor) where a prisoner escape (tumor cell invades systemic vasculature) initiates an acute reaction involving armed patrol forces (cytotoxic T cells) for the order to be resumed.

Adjuvant or neo-adjuvant chemotherapy for STS in general is a point of debate [32, 5]. Risk stratification of LMS could assist in selecting patients who might benefit from adjuvant treatment. Our initial results in primary LMS tumors suggest that patients with positive CD8 and PD-L1 staining have a poor prognosis and as such might benefit from intensified adjuvant/neoadjuvant therapy. In addition to prognosis there is some evidence from other neoplasms that PD-L1 staining may also be predictive for response to therapy. In breast cancer for example PD-L1 predicted complete pathological response to neoadjuvant chemotherapy [33] thus it merits further investigation whether LMS with positive PD-L1 staining are not only in need for adjuvant therapy but also more likely to benefit from it.

The limitations of our study lay in its small size therefore we are planning to confirm the prognostic role of PD-L1 expression and CD8 infiltration in a larger LMS cohort. A further point is the discrepancy between the two PD-L1 antibodies tested.

In summary, in this pilot we found that immune-phenotyping of LMS might allow risk stratification of patients directing therapeutic decisions regarding chemotherapy as well as identifying patients likely to benefit from immune-checkpoint inhibitors. Furthermore, characterizing the tumor microenvironment of LMS might point to combined approaches to facilitate an immune-response such as immune-checkpoint inhibitors with metabolic factors (e.g. Indoleamine-pyrrole 2,3-dioxygenase (IDO1) inhibitors), targeted therapy (PI3K-AKT inhibitors) and other strategies. Multi-center studies will be necessary in order to allow large scale analysis and collection of sufficient data to allow firm conclusions and subsequent trials to test effectiveness.

## MATERIALS AND METHODS

### Study population and clinical data

We identified 11 patients with LMS treated and followed at The Sharett Institute of Oncology, Hadassah Medical Center. All these patients had signed an institutional informed consent which permits comprehensive tumor profiling (IRB 346-12 HMO). For the identified cases archived tissue slides were retrieved and the histological diagnosis of LMS was confirmed by a board-certified Pathologist (Eli Pikarsky). Data on date and stage of diagnosis, recurrences and survival were collected from patient's electronic file.

### Immunohistochemistry

Paraffin embedded formalin fixed tissue blocks of primary LMSs were retrieved from the pathology archive and microtome sliced to prepare sections for immune-staining.

Mismatch-repair status was determined via staining for the proteins: MLH1 (Mouse Monoclonal Primary Antibody, cat#. 790-4535), PMS2 (Rabbit Monoclonal Antibody, cat#. 760-4265), MSH6 (Mouse Monoclonal Primary Antibody, cat#. 790-4455) and MSH2 (Rabbit Monoclonal Antibody, cat#. 760-4531). Secondary antibody detection was carried out by using OptiView DAB IHC Detection Kit cat#. 760-700; all reagents and antibodies were obtained from Ventana Medical Systems (Tucson, AZ).

Immune-infiltrate was analyzed using a pan-T cell marker anti-CD3 (1:150, clone 103A-76, CellMarque, catalog# 1507011D), and anti-CD8 (1:50, clone C8/144B, CellMarque, catalog# 108M-96) was utilized to identify cytotoxic T cells. The expression of the lymphocyte activation marker programmed cell death 1 (PD-1) was determined with anti-PD-1 (1:25, clone NAT105, CellMarque, catalog# 315M-95). The expression of the PD-1 inhibitory ligand PD-L1 was initially assessed with an antibody from Abcam (1:50, Abcam, catalog# ab205921) then repeated via an antibody adopted for clinical use (Dako kit, clone 22C3) we employed normal tonsil and normal placenta as positive controls. Photomicrographs were taken with an Olympus light microscope and acquisition system.

Staining for all antibodies was performed with an automated Benchmark XT machine.

Immuno-stained slides were examined by a board certified senior pathologist (Eli Pikarsky). Staining intensity on a scale of 0 to 3 and percentage of positive cells (for PD-1) were determined within the tumor as well as at the periphery of the tumor. A cutoff of >1% was used to define PD-L1 positivity.

### Statistical analysis

The association between outcome (a dichotomous variable) and TIL, PD-L1 and mismatch-repair (MMR) protein expression (ordinal variables), was tested by using the Fisher's exact test and the non-parametric Mann-Whitney test. The tests were two-tailed, and a *p*-value of 5% or less was considered statistically significant.

## Abbreviations

LMS: Leiomyosarcoma
TIL: tumor lymphocytic infiltration
PD-L1: programed cell death ligand-1
MMR-D: mismatch repair deficiency
ULMS: uterine LMS
STS: soft tissue sarcomas
NSCLC: non-small cell lung cancer
LMS: EU-LMS extra-uterine
IDO: indoleamine-pyrrole 2,3-dioxygenase
